# Infliximab Preferentially Induces Clinical Remission and Mucosal Healing in Short Course Crohn's Disease with Luminal Lesions through Balancing Abnormal Immune Response in Gut Mucosa

**DOI:** 10.1155/2015/793764

**Published:** 2015-03-19

**Authors:** Lijuan Yu, Xuehua Yang, Lu Xia, Jie Zhong, Wensong Ge, Jianxin Wu, Hongchun Liu, Fei Liu, Zhanju Liu

**Affiliations:** ^1^Department of Gastroenterology, The Shanghai Tenth People's Hospital of Tongji University, Shanghai 200072, China; ^2^Department of Gastroenterology, Ruijin Hospital of Shanghai Jiaotong University, Shanghai 200025, China; ^3^Department of Gastroenterology, Xinhua Hospital of Shanghai Jiaotong University, Shanghai 200092, China; ^4^Department of Gastroenterology, Zhongshan Hospital of Fudan University, Shanghai 200032, China; ^5^Department of Gastroenterology, East Hospital of Tongji University, Shanghai 200120, China

## Abstract

This study was undertaken to evaluate the efficacy of infliximab (IFX) in treatment of Crohn's disease (CD) patients. 106 CD patients were undergoing treatment with IFX from five hospitals in Shanghai, China. Clinical remission to IFX induction therapy was defined as Crohn's disease activity index (CDAI) < 150. Clinical response was assessed by a decrease in CDAI ≥ 70, and the failure as a CDAI was not significantly changed or increased. Ten weeks after therapy, 61 (57.5%) patients achieved clinical remission, 17 (16.0%) had clinical response, and the remaining 28 (26.4%) were failed. In remission group, significant changes were observed in CDAI, the Simple Endoscopic Score for Crohn's Disease (SES-CD), and serum indexes. Patients with short disease duration (22.2 ± 23.2 months) and luminal lesions showed better effects compared to those with long disease duration (71.0 ± 58.2 months) or stricturing and penetrating lesions. IFX markedly downregulated Th1/Th17-mediated immune response but promoted IL-25 production in intestinal mucosa from remission group. No serious adverse events occurred to terminate treatment. Taken together, our studies demonstrated that IFX is efficacious and safe in inducing clinical remission, promoting mucosal healing, and downregulating Th1/Th17-mediated immune response in short course CD patients with luminal lesions.

## 1. Introduction

Crohn's disease (CD) is a chronic relapsing and remitting inflammatory disorder of any part of the gastrointestinal tract. To date, the incidence and prevalence of CD are increasing in China [1, 2]. Although accumulating evidence indicates that CD is the consequence of a dysregulation of innate and adaptive immune responses to commensal enteric bacteria in a genetically susceptible host, the etiology of the disease still remains elusive [3, 4]. It has been shown that T helper cell (Th) 1 related proinflammatory cytokines (e.g., TNF, IFN-*γ*) and Th17-associated cytokines (e.g., IL-17A, IL-21, and IL-23) are significantly increased, but IL-25 is markedly decreased in the inflamed mucosa of CD patients [5–7]. Traditional treatments, including 5-aminosalicylates, enteral nutrition, corticosteroids, and immunosuppressive agents, have unsatisfied clinical outcomes in some patients, and some may rely on corticosteroids or increase their risk of developing steroid-related adverse effects on the basis of a long-term treatment. Therefore, it is imperative that other therapeutic options are considered. In recent years, although several biologic drugs, best represented by anti-TNF mAb (infliximab, IFX), have been developed in inducing remission in CD patients, their advent has revolutionized the disease treatment [8].

IFX is a monoclonal IgG1 antibody targeted against antitumor necrosis factor (TNF), which is composed of a human constant region IgG1 light chain that accounts for nearly 75% of the antibody and a mouse variable region (25%). The main mechanism of IFX is to neutralize the biological activity of TNF by binding with high affinity to the soluble and transmembrane forms of TNF and to inhibit binding of TNF with its receptors (p55/p75 subunits) [9], which came up with a new approach for the treatment of active CD with very inspiring results in the field of efficacy and safety [10]. Moreover, other mechanisms also play a role, ever not fully understood, in affecting barrier function, ADCC activation, lymphocyte apoptosis, mucosal angiogenesis, and regulating inflammatory cytokines in intestinal mucosa [11].

IFX is the first biologic agent approved for the treatment of CD. Previous work has shown that IFX is more effective than placebo in randomized controlled trials at inducing remission of active CD, maintaining remission of the disease, and promoting mucosal healing and fistula closures in CD patients [9, 12–14]. Moreover, evidences have also demonstrated that IFX therapy has striking response and remission rates, decreases CD-related hospitalization and the rate of surgery, improves the quality of life, and reduces the costs of care for CD patients without an increase in side reactions [15].

In this study we found that IFX was effective in inducing clinical remission and promoting intestinal mucosal healing in CD patients, particularly in those short course patients with luminal lesions, while the failure of IFX therapy was frequently observed in CD patients with long disease duration or stricturing and penetrating lesions. Furthermore, IFX could markedly suppress Th1/Th17-associated proinflammatory cytokine production and upregulate IL-25 expression in inflamed mucosa of CD patients.

## 2. Materials and Methods

### 2.1. Ethics Statement

These retrospective studies were approved by the Shanghai Tenth People's Hospital of Tongji University, Shanghai, China; Ruijin Hospital of Shanghai Jiaotong University, Shanghai, China; Xinhua Hospital of Shanghai Jiaotong University, Shanghai, China; Zhongshan Hospital of Fudan University, Shanghai, China; and East Hospital of Tongji University, Shanghai, China, from December 2009 to October 2013. The Institutional Review Board and Ethics Committee at each study center approved the protocol, and all patients provided written informed consent. All authors had access to the study data and had reviewed and approved the final paper. Eligible patients had an established diagnosis.

### 2.2. Patient and Sample Collection

One hundred and six patients with CD who had been treated with IFX were recruited in five university hospitals in Shanghai, China. The diagnosis of CD was based on conventional clinical features and radiological and endoscopic features, and finally confirmed by histological examination of ileal and colonic biopsies [16]. Cases were determined according to the Montreal classification system [17]. They were all naive to biological agent therapy and received IFX at a dose of 5 mg/kg body weight at weeks 0, 2, and 6 as an IFX induction regimen. It was administered by a two-hour intravenous infusion. The transfusion reaction was monitored at the same time. Laboratory parameters such as C-reactive protein (CRP), erythrocyte sedimentation rate (ESR), hemoglobin (Hb), and albumin (Alb), as well as clinical data such as Crohn's disease activity index (CDAI, calculated as defined by Best et al. [18] which was filled by physicians), adverse reactions, and occurrence of complications, were monitored at time of registration and at each follow-up visit. Colonoscopy was performed prior to and 10 weeks after initial treatment of IFX, and mucosal ulceration status was assessed by the Simple Endoscopic Score for Crohn's Disease (SES-CD) score 0 to 3 of every five ileocolonic segments under ileocolonoscopy. Endoscopic remission was determined as a SES-CD score of 0 to 2 [19]. Intestinal biopsies were taken at sites of active inflammation adjacent to ulcerations for histology and analysis of the mRNA levels of Th1 related cytokines (TNF, IFN-*γ*), Th17 related cytokines (IL-17A, IL-21, IL-23p19, and RORC), IL-25, and IL-10, which were associated with CD pathogenesis by quantitative real-time polymerase chain reaction (PCR).

### 2.3. Definition of the Efficacy of IFX

The clinical efficacy of IFX in our study was evaluated at week 10 after initial administration, the point at which patients were followed up for sustaining treatment of IFX. Clinical remission was defined as a CDAI score of <150 points, and clinical response as a decrease in the CDAI score ≥ 70 points at the evaluation time point compared to the baseline index. The failure category included all the remaining patients, whose CDAI was not significantly changed or increased [16, 18, 20].

### 2.4. Evaluation of Safety

Any adverse events happening during the treatment were recorded, including infusion reactions or adverse events believed to be associated with IFX. Infusion reaction was defined as any adverse event occurring during an infusion or within 1 to 2 hours after the infusion like fever, chills, primarily chest pain, dyspnea, pruritus, and urticaria. Anaphylaxis might occur at any time during IFX infusion. Severe adverse events were defined as serious adverse events resulting in death, life threatening, requiring hospitalization, or persistent or significant disability.

### 2.5. Quantitative Real-Time PCR

Total RNA was extracted from the fresh-frozen biopsies using the RNeasy Kit (Invitrogen Life Technologies, Grand Island, NY, USA) according to the manufacturer's instructions. Total RNA quantity and quality isolated from each sample were assessed using a NanoVue spectrophotometer (GE Healthcare, Piscataway, NJ, USA), with a 260/280 ratio of >1.8 and 28S/18S ratio of >1.4 for the majority of the samples. The cDNA was synthesized with SYBR PrimeScript RT reagent kit (TaKaRa, Dalian, China) according to the manufacturer's instructions. Quantitative real-time RT-PCR was performed in the ABI prism 7900 HT sequence detector (Applied Biosystems, Foster City, CA, USA) using SYBR green methodology. *β*-Actin was used as the endogenous reference gene. All primers were synthesized and purchased from Sheng Gong BioTech (Shanghai, China) and used according to standard methodologies. All PCR reactions were run in triplicate and performed with 40 cycles using the following conditions: 95°C for 1 min, followed by 40 cycles at 95°C for 15 sec and 60°C for 30 sec. The relative target gene expression levels were calculated as a ratio relative to the *β*-actin reference mRNA. Quantitative real-time PCR analysis was carried out using the 2^−ΔΔCt^ method [21–23].

### 2.6. Statistical Analysis

Data are expressed as mean ± standard deviation or percentage. The baseline characteristics of the patients classified into clinical remission, clinical response, and failure groups were estimated by a simple descriptive analysis and the *χ* ^2^ test. Parameters including CDAI, CRP, ESR, Hb, Alb, SES-CD, and inflammatory cytokines were compared with Student's *t*-test for quantitative variables. Statistical analysis was performed using SPSS Statistics version 16.0 (SPSS, Chicago, IL, USA). A value of *P* < 0.05 was considered to be statistically significant.

## 3. Results

### 3.1. Baseline Demographics

As shown in Table 1, a total of 106 CD patients (77 males and 29 females) were recruited in our study to receive IFX therapy with CDAI ranging from 153 to 519. The mean age of diagnosis was 27 years old (13–78 years old) and mean duration was 38.68 months (0.5–192 months) before starting treatment. Eighty-five (80.2%) patients were diagnosed from 17 to 40 years of age (A2); 66 (62.3%) of them had ileocolonic disease (L3, both ileal and colonic involvement) and no patients had isolated upper gastrointestinal disease (L4). Forty-nine (46.2%) had an initial nonstricturing and nonpenetrating behavior of diseases (B1). Perianal diseases were present at 32 (30.2%) patients before treatment, and 28 of them had perianal fistula. Only 19 (17.9%) of the patients were current smokers. Twelve patients (11.3%) had prior bowel resection for CD. There were 49 (46.2%) never receiving traditional therapy such as corticosteroids or immunosuppressive agents called “top-down strategy,” while the so-called “step-up strategy” is based on the traditional therapeutic approach of CD, progressive intensified course of treatment, as the disease severity increases. Both the “top-down strategy” and the “step-up strategy” patients received combination of immunosuppressive agents (e.g., azathioprine), which was most commonly used in each referred center. For the fistulae patients routine antibiotics (metronidazole and/or ciprofloxacin) were also administrated.

### 3.2. Efficacy of IFX in the Treatment of CD

This study included 106 active CD patients with a median CDAI score of 223 (range from 153 to 519) prior to starting therapy. Significant decrease was observed in the mean values of CDAI when compared with data at baseline and after treatment (233.0 ± 69.1 versus 148.5 ± 80.2; *P* < 0.05), as well as the levels of serum CRP, ESR, Hb, Alb, and SES-CD (*P* < 0.05). Based on the change of CDAI, 61 (57.5%) participants achieved clinical remission with CDAI below 150, another 17 (16.0%) did not squeeze into “clinical remission,” but they were up to the standard “clinical response” with a decrease of CDAI ≥ 70, but ≥150. The remaining 28 patients (26.4%) were unfortunately classified as failure to IFX with both CDAI ≥ 150 and a decrease of CDAI ≤ 70 or an increase of CDAI from the the baseline.

Table 2 shows the demographic database of three groups throughout the study period. Twenty-eight patients in failure group were composed of 18 (64.3%) “step-up strategy” and 10 (35.7%) “top-down strategy” patients. There were no apparent differences in age of diagnosis or sex, but patients in failure group had a significantly longer duration of disease compared with those in remission group (*P* < 0.01). A statistical difference was also observed in patients with three kinds of disease behaviors (B1, B2, and B3) according to the Montreal classification by chi-square test, showing that IFX appeared to be more effective in CD patients with B1 behavior than in those with B2 and B3 behaviors (*P* < 0.01). Moreover, no differences were observed among CD patients according to disease locations (*P* > 0.05).

**Figure 1:**
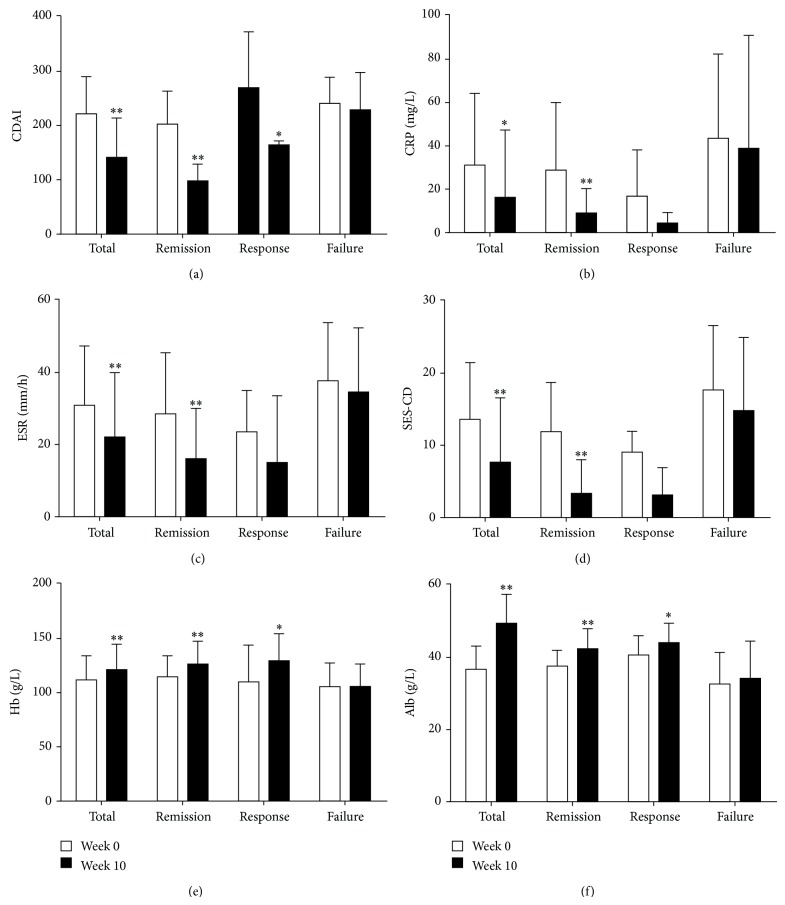
IFX therapy induces clinical remission and promotes intestinal mucosal healing in CD patients. The changes of CDAI (a), CRP (b), ESR (c), SES-CD (d), Hb (e), and Alb (f) were analyzed at weeks 0 and 10, respectively. ^*^ *P* < 0.05, ^**^ *P* < 0.01 versus values before IFX therapy.

Figure 1 shows the changes of active indices of CD patients from three groups described above. All serum indices were significantly improved at week 10 after the commencement of treatment (*P* < 0.05). Serum indices such as CRP and ESR were markedly decreased in remission group than in clinical response and failure groups (*P* < 0.05). Likewise, serum Hb and Alb were remarkably increased in remission group than in clinical response and failure groups (*P* < 0.01). In contrast, no significant changes were observed in failure group during the study period.

### 3.3. Mucosal Healing after IFX Therapy

**Figure 2:**
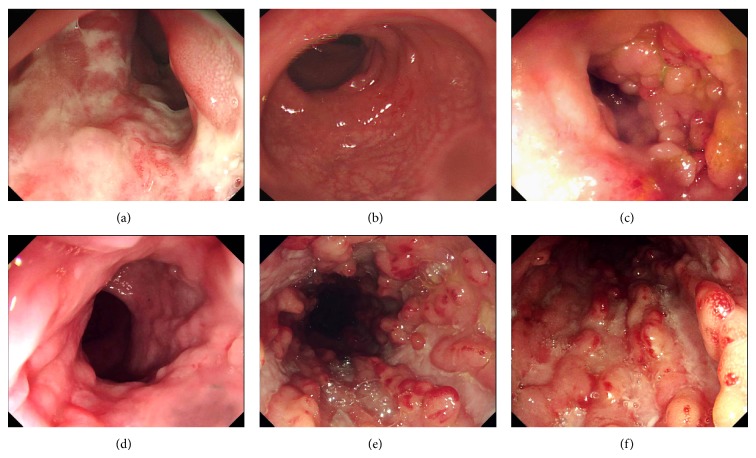
IFX therapy promotes intestinal mucosal healing in CD patients. Representative endoscopic photographs are demonstrated from a patient in remission group (a, b), a patient from response group (c, d), and a patient from failure group (e, f) before (a, c, and e) and 10 weeks after IFX treatment (b, d, and f).

To evaluate intestinal mucosal healing after IFX therapy, all patients underwent endoscopy before and 10 weeks after IFX induction therapy. As shown in Figures 1 and 2, SES-CD was found to be significantly decreased 10 weeks after IFX therapy compared with that before therapy in all patients (13.6 ± 7.7 versus 7.75 ± 8.7; *P* < 0.05). Surprisingly, the mean values of SES-CD from CD patients in remission group were markedly decreased 10 weeks after TNF administration compared to those before IFX treatment (7.7 ± 7.0 versus 2.9 ± 5.3; *P* < 0.01). Of note, 28 patients (26.4%) got endoscopic remission, 20 patients (18.9%) were in deep remission (both SES-CD ≤ 2 and CDAI < 150), 7 (6.6%) were from clinical response group, and the only 1 left was from failure group.

### 3.4. Cytokines Profiles after IFX Therapy

Intestinal biopsies were taken from 53 patients including 25 in remission group, 15 in response group, and 13 in failure group. As shown in Table 3, the mRNA levels of Th1-associated cytokines (TNF, IFN-*γ*) and Th17-associated cytokines (IL-17A, IL-21, IL-23p19, and RORC) were found to be markedly decreased 10 weeks after IFX therapy as compared with those before IFX treatment in remission group (*P* < 0.005), but IL-25 was significantly increased (*P* < 0.005) as compared with that before IFX treatment. Likewise the active indices of CD, no significant changes were observed in the failure group before and 10 weeks after IFX treatment. IL-10 was found to be not statistically significant in all three groups. These data suggest that IFX induces mucosal healing through downregulating Th1/Th17-associated cytokines and promoting IL-25 production, thus balancing abnormal immune responses in intestinal mucosa.

### 3.5. Outcome of Fistula

Table 4 shows the outcomes of fistulae after IFX therapy which were estimated by endoscopic or magnetic resonance imaging (MRI). In our study, 46 CD patients were recorded to have fistulae including perianal, enterocutaneous, enterovaginal, and intestinal fistulae. The efficacy of IFX therapy in the CD patients with fistulae was observed to have a significant difference by chi-square test, showing that the percentage of fistulae closure/improvement in remission and response groups (75.0%) was significantly higher than that in failure group (11.1%) after IFX therapy (*P* < 0.01). It was worth noting that 17 patients (60.7%) with perianal fistula had good response to IFX therapy, showing closure/improvement of fistula. However, among 11 patients (39.3%) with IFX therapy failure, the majority of participants (63.6%) were from the failure group. Interestingly, poor efficacy was observed in CD patients with enterovaginal and intestinal fistulae, particularly from the failure group. Notably, three patients with enterocutaneous fistulae recovered entirely.

### 3.6. Adverse Effects

Twenty-four patients (22.6%) experienced adverse events, and none of them had serious adverse event and discontinued therapy during the induction phase (Table 5). The most common reactions were rash or fever belonging to infusion reactions, which were observed in 8 episodes and 7 cases at or after the injection site separately while another 2 patients developed mild elevations of ALT (85 and 72 U/L, resp., normal reference value: 0–40 U/L) after first IFX injection and showed no symptoms. Patients who suffered from adverse events were successfully treated with conventional medical therapy and it had no effect on the subsequent progress. Taken together, IFX therapy was well tolerated in active CD patients even though it was a short-term follow-up.

## 4. Discussion

The treatment of CD is aimed at inducing remission, maintaining remission, preventing relapses, controlling complications, restoring intestinal physiological function, and postponing surgical intervention. IFX has been reported to bring about expectations with the main mechanism of blocking the role of TNF in the treatment of IBD. In the current study, we reported the efficacy of IFX in inducing clinical remission and promoting mucosal healing in active CD patients from multicenter of China. We demonstrated a response to IFX induction therapy in 73.6% and intestinal mucosa benefit achieving endoscopic remission under endoscopic examination in 26.8% CD patients. In addition, 57.5% of CD patients got CDAI scores < 150 at the last time point of follow-up, indicative of remission. Furthermore, proinflammatory cytokines (e.g., TNF, IFN-*γ*, IL-17A, IL-21, and IL-23) were markedly downregulated, while expression of IL-25 was highly upregulated in inflamed mucosa of CD patients after IFX treatment in remission group. These data indicate that short course CD patients with luminal lesions without serious complications preferentially achieve clinical remission or response.

Previous work has indicated that early treatment with IFX is usually associated with better response, reducing intestinal mucosal lesions, preventing disease progression, and avoiding complications by comparing three groups of CD patients with disease duration of 2 weeks, 2 months, and 7.5 years, respectively [12, 20, 24–27]. Study by Johnson et al. [28] also reported that early treatment could reduce tissue fibrosis by histological scoring of fibrosis at day 21 from the* Salmonella typhimurium* mouse model of intestinal fibrosis. In our study, we observed that patients with shorter duration of disease accounted for the most in clinical remission by IFX administration compared with parallel with longer disease period. Furthermore, our study also showed that the patients with luminal inflammatory disease had significantly better clinical benefit than those with stricturing or penetrating disease, consistent with the previously reported [29]. It likely suggests that patients with severe diseases such as stricture or penetration may need alternative therapeutic approaches, such as surgical or endoscopic interventions.

In our study, the indices reflecting CD activities (e.g., CRP, ESR, Hb, Alb, and SES-CD ) were significantly improved in remission group after IFX therapy (Figure 1), consistent with previous reports [30, 31]. Hb and Alb as the parameters reflecting the nutritional status were apparently improved after IFX treatment. It is hypothesized that IFX therapy could restore intestinal epithelial barrier, leading to enhanced absorption of nutrients and iron. In addition, patients with perianal fistulae have better outcomes than intestinal fistulae. IFX is reported to block physiological binding of TNF and reduce tissue inflammation [32]. We suspect that perianal fistulae do not have more chances to intimately contact intestinal flora leading to local inflammatory response, while intestinal fistulae contact intestinal flora continuously, even if TNF is suppressed. Moreover, other proinflammatory mediators may be also sustained. For these patients with a failure of IFX therapy, the more frequencies of complex or aggressive disease phenotypes are present, and other therapeutic approaches (e.g., surgery, novel biological agents) may be warranted.

Our data have shown that IFX therapy could downregulate Th1/Th17-associated proinflammatory cytokines and promote IL-25 production, consistent with previous work showing that serum level of IL-23 was significantly decreased in rheumatoid arthritis patients treated with IFX [33]. Even though IL-25 is structurally related with Th17 cytokine family, it is not only necessary for the induction of Th2-mediated immune response [34], but also required for the generation of innate type cells which may produce Th2-associated cytokines at the initiation of an adaptive Th2-mediated response [35]. Our recent work has proved that IL-25 markedly suppresses IBD CD4^+^ T-cell activation to produce proinflammatory cytokines [31]. Taken together, the induction of clinical remission and promotion of intestinal mucosal healing in active CD patients by IFX may be owing to downregulating the expression of proinflammatory cytokines (e.g., IL-17A, IL-21, and IL-23).

Previous works have shown that the CD patients who encountered failure or infusion reactions may be related to the formation of antibodies to IFX (ATI) [36, 37]. Furthermore, a direct relationship between the failure to IFX and the presence of ATIs has been confirmed in serum samples levels during IFX induction phase [38]. Therefore, it is necessary to monitor serum ATI concentrations, which can be used to predict response or infusion reactions. It is also helpful to adjust the dosage, particularly the trough level of IFX, for individual administration to improve efficacy.

Several mild side effects have been observed during IFX infusion, such as fever, chill, rash, pruritus, and dyspnea. No severe adverse events were seen in our study, which is different from earlier reports of infections, malignancy, and deaths [37, 39, 40]. The reactions are attributed to systemic immune response, susceptibility genetic background, or environment factors.

## 5. Conclusions

In summary, our experience presented here details the efficiency of IFX in the management of CD patients from multicentral study. Patients are more likely to achieve clinical benefit if they have luminal inflammatory disease and short disease duration. IFX downregulates Th1/Th17-associated proinflammatory cytokines and upregulates IL-25 expression in intestinal mucosa. We have also demonstrated a good safety profile with IFX, albeit during a short follow-up period. However, the effect on intestinal fistulae patients was far from satisfactory.

## Figures and Tables

**Table 1:** Characteristics of patients with CD in our study.

Gender (M/F)	77/29
Mean duration of disease before IFX in months	38.7 ± 42.9
Mean age of diagnosis in yrs	27.2 ± 9.7
A1 (≤16 yrs)	10 (9.4%)
A2 (17–40 yrs)	85 (80.2%)
A3 (>40 yrs)	11 (10.4%)
Location	
L1 (ileum only)	18 (17.0%)
L2 (colon only)	22 (20.8%)
L3 (ileocolonic)	66 (62.3%)
L4^*^	0
Behavior	
B1 (nonstricturing, nonpenetrating)	49 (46.2%)
B2 (stricturing)	29 (27.4%)
B3 (penetrating)	28 (26.4%)
*P* ^#^	32 (30.2%)
Fistula	
Perianal	28 (26.4%)
Enterocutaneous	3 (4.8%)
Enterovaginal	3 (2.8%)
Intestinal	12 (11.3%)
Current smoker	19 (17.9%)
Previous CD-related abdominal surgery	12 (11.3%)
Step-up/top-down strategy	57/49

^*^A modifier that can be added to L1–L3 when concomitant upper gastrointestinal disease is present.

^#^Added to B1–B3 when concomitant perianal disease is present.

**Table 2:** Demographic databases of patients in clinical remission, clinical response, and failure after IFX induction therapy.

Variable	Remission group (*n* = 61)	Response group (*n* = 17)	Failure group (*n* = 28)	*P* value
Age of diagnosis, yrs	27.3 ± 8.5	25.6 ± 9.4	28.0 ± 12.4	0.72
A1 (≤16)	5	2	3	0.99
A2 (17–40)	50	13	22	
A3 (>40)	6	2	3	
Gender, male/female	46/15	10/7	21/7	0.38
Disease duration, mths	22.2 ± 23.2 (0.5–96)	44.6 ± 37.9 (3–147.6)	71.0 ± 58.2 (2–192)	<0.0001
Location				
L1, *n* (%)	11 18.0	4 23.5	3 10.7	0.03
L2, *n* (%)	7 11.5	7 41.2	8 28.6	
L3, *n* (%)	43 70.5	6 35.3	17 60.7	
Behavior				
B1, *n* (%)	38 62.3	5 29.4	6 21.4	0.0007
B2, *n* (%)	15 24.6	6 35.3	8 28.6	
B3, *n* (%)	8 13.1	6 35.3	14 50.0	
Current smokers, *n* (%)	8 13.1	4 23.5	7 (25)	0.32
Step-up strategy, *n* (%)	26 42.6	13 76.4	18 64.3	0.02
Top-down strategy, *n* (%)	35 57.4	4 23.6	10 35.7	

**Table 3:** Changes of inflammatory cytokines in inflamed mucosa of CD patients before and 10 weeks after IFX treatment.

		Remission group (*n* = 25)	Response group (*n* = 15)	Failure group (*n* = 13)
IFN-*γ*	Week 0	112.6 ± 34.5	118.9 ± 32.1	117.2 ± 30.4
	Week 10	32.5 ± 11.2^*^	64.7 ± 21.6^*^	109.2 ± 32.6

TNF	Week 0	62.3 ± 12.5	63.8 ± 13.2	61.8 ± 15.8
	Week 10	11.5 ± 4.5^*^	40.1 ± 8.9^+^	58.9 ± 14.7

IL-10	Week 0	8.5 ± 3.6	9.8 ± 3.9	9.2 ± 3.5
	Week 10	12.8 ± 2.9	12.5 ± 4.1	8.9 ± 3.0

IL-17A	Week 0	35.8 ± 10.5	34.6 ± 9.8	36.5 ± 8.7
	Week 10	7.6 ± 3.2^*^	18.8 ± 5.2^*^	36.7 ± 10.1

IL-21	Week 0	21.3 ± 6.3	20.1 ± 7.2	21.4 ± 6.1
	Week 10	3.6 ± 1.2^*^	11.9 ± 3.5^*^	19.8 ± 5.9

IL-23p19	Week 0	24.5 ± 6.8	25.5 ± 5.4	26.9 ± 5.7
	Week 10	5.8 ± 2.1^*^	12.8 ± 4.3^+^	24.5 ± 6.6

IL-25	Week 0	8.2 ± 3.2	9.8 ± 3.6	7.9 ± 3.5
	Week 10	26.9 ± 8.3^*^	15.6 ± 4.8	8.8 ± 2.9

RORC	Week 0	26.1 ± 7.0	29.4 ± 8.8	25.8 ± 7.3
	Week 10	7.6 ± 3.5^*^	16.7 ± 6.7^*^	23.4 ± 5.7

^*^ *P* < 0.005; ^+^ *P* < 0.05 versus values before initial therapy with IFX.

**Table 4:** Efficacy of IFX therapy on fistula of CD patients.

Variable	*n*	Remission group (failed/total)	Response group (failed/total)	Failure group (failed/total)
Perianal	28	3/15	1/5	7/8
Enterocutaneous	3	0/2	0/1	0/0
Enterovaginal	3	0/1	1/1	1/1
Intestinal^a^	12	0/1	2/2	8/9

Total^b^	46	3/19	4/9	16/18

^a^Two patients with intestinal fistula also had a perianal fistula.

^b^ *P *< 0.01 by chi-square test.

**Table 5:** Adverse effects during IFX therapy in CD patients.

Description of the events	Number of patients (%)
Any adverse events	24 (22.6%)
Any serious adverse events	0
Infusion reactions	22 (20.8%)
Fever or chills	7 (6.6%)
Primarily chest pain	0
Dyspnea	0
Myalgia	4 (3.8%)
Pruritus/rash	8 (7.5%)
Nausea/vomiting	3 (2.8%)
Anaphylaxis	0
Seizures	0
Hypotension	0
Infections	0
Hepatotoxicity	2 (1.9%)
Lupus-like syndrome	0
Psoriasiform rash	0
Deaths	0
